# Fundamental Themes in Social–Emotional Learning: A Theoretical Framework for Inclusivity

**DOI:** 10.3390/ijerph21040506

**Published:** 2024-04-19

**Authors:** Mickayla Dussault, Robert B. Thompson

**Affiliations:** Psychology Department, University of Southern Maine, Portland, ME 04103, USA; rbthompson@maine.edu

**Keywords:** social–emotional learning, trauma-informative care, public mental health, social development, equitable education

## Abstract

Social–emotional learning (SEL) is a rapidly growing field of research that has garnered significant attention in recent years. Each facet of SEL research in fields such as education, mental health, and developmental research has used specific methodologies and terms in their narrow research focus. In education specifically, where the most SEL research has been produced, many frameworks have implementation requirements. The lack of a framework focused on overarching themes without implementation requirements prevents the fields from coming together to compile and compare research and progress to create parent-, adult-, or mental health-specific SEL programs. This paper provides a conceptual analysis of SEL, aimed at clarifying the concept and deconstructing its various facets. This framework is needed to acknowledge the many different terms and skills for the same principle while also narrowing down definitions for clarity. The resulting framework can be used as a basis for future research, practice, and policy discussions in the field.

## 1. Introduction

Social–emotional learning (SEL) in schools is not a new concept, despite its relatively new name. Plato believed that good citizenship, social justice, and bolstering a student’s natural talents should be the focus of education. He also believed that a society that supported its children would in turn create people that wanted to support society [1]. SEL and its place in formal education has been debated for thousands of years. Although the importance of social and emotional wellbeing for overall development has been recognized for centuries, the underlying cognitive and emotional constructs and specific skills are still highly debated and discussed, particularly as it impacts educational policy and SEL curriculum. This narrative review of the literature is intended to help clarify terminology and provide a synthesis of the dominating perspectives and areas of disagreement, while also discussing key areas of cognitive developmental neuroscience that are often left out of debates around SEL principles and educational best practices.

With the rise of psychoanalysis in the early 1900s came a general focus on childhood experiences, child rearing, and education. The works of Piaget, Wall, Montessori, Aichorn, Vygotsky, and many others rose to great prominence to advocate for and debate how the public can support children through SEL and education. In the new millennium, SEL programs, approaches, and research have had an upswing in interest due to concerns regarding children’s mental health. More specifically, there is abundant research supporting the argument that emotional regulation and wellbeing are prerequisites for key areas of academic and intellectual functioning, especially around abilities related to executive function and metacognitive processes. Mental health struggles and feelings of isolation, particularly during the COVID-19 pandemic, have affected physical health, the economy, and the community [2,3]. There are now over one hundred years of data on SEL and social–emotional skills rather than just theory and debate. An EBSCOhost search for “social–emotional learning” in the ERIC database alone retrieves over 3000 articles.

Mental health challenges are well-documented consequences of poverty. For example, in national data from Scotland, 4-year-olds who lived in impoverished households had over double the amount of disruptive behaviors and emotional difficulties compared to their peers who lived in affluent areas [4]. The connection between mental health and poverty also extends beyond children. Parents are more likely to mistreat or neglect their children when they have impoverished backgrounds. This could be due to stress, working long hours, not having much time with the child, or not having access to food, clean water, and housing. Parents who receive welfare resources after being reported to Child Protective Services (CPS) are less likely to reoffend [5,6]. A separate study found that only 5.7% of parents who were reported to CPS reoffended after attending a parenting support program that included teaching social–emotional skills (Project Support) compared to 27.7% who reoffended in the control group [7]. Similar patterns can be found in research regarding at-risk parents’ participation in SEL programming [8].

Underlying social issues such as poverty and abuse have been documented to increase the risk of social–emotional difficulties. This includes emotional and behavioral challenges in both children and parents. Like many social/economic preventative measures, enacting a social–emotional learning (SEL) program may be far less expensive over time than welfare and may reach rural areas [9], which typically lack resources but have access to a school. Including SEL programming in school settings may set up the child to be a better parent in adulthood despite their income background.

Both scientists and politicians are looking to prevent or mitigate increasing rates of mental illness, problem behaviors, and social challenges in society; SEL appears to be at the nexus of recent literature [10,11,12] However, the construct of SEL is promising for the same reason that it is challenging; it encompasses all social and emotional skill building. While social–emotional learning is recognized as an umbrella term for learning any skills with both social and emotional goals [13], there is confusion and debate as to what skills make up social–emotional learning, what the difference between programs or approaches are, and how they compare against one another. These conceptual challenges deeply impact implementation efforts. The implementation of SEL support in education settings requires the involvement of parents. The implementation of SEL through agencies like CPS, which are in a position to advocate for SEL support in parenting programs, requires political leadership. The implementation of SEL in mental health settings requires consistent understanding from social workers and mental health professionals. Unfortunately, these agencies, disciplines, and advocacy groups often work within very different contexts of care and management and are often not using the same constructs or nomenclature in their discussion of SEL. For these reasons, the goal of this paper is to clarify SEL as a concept, deconstruct its various facets, and create a way to meaningfully compare SEL theories that can be understood by researchers and parents alike. Therefore, the primary objective of this paper is to provide a conceptual taxonomy that specifies how social–emotional skills are defined within the diverse scholarly literature. In particular, we aimed to identify areas where differences in terminology may occur but refer to the same or highly overlapping constructs. Another key goal is to further integrate neurocognitive and developmental perspectives that relate to SEL in order to better bridge gaps between psychological theory and the SEL implementation literature. As such, this review is explicitly not intended to specify or recommend particular ways to implement SEL supports and curricula, but rather to help clarify how social–emotional skills are learned to help researchers and practitioners approach the topic in a more inclusive, contextually sensitive, and organized way.

### Importance of a Framework

When a person says “social–emotional learning” it can mean one of three things. It can mean the learning of social–emotional skills as part of normative development, the learning outcomes within a specific program, or the learning of skills within a general approach. An SEL *approach* is a broad pedagogical paradigm, or theory of teaching. The difference between a *program* and an *approach* is that the first is much more regulated, with resources, teacher supports, and specific activities, while the latter is a more general set of theories and beliefs. A program can reflect an approach, taking on the methods and beliefs and applying them within specific activities. An approach cannot be a program, since it is broad. There is also the fact that SEL is not just an education-focused issue; social–emotional skill programs could also be helpful in parent classes through CPS, mental health settings, and conversations on diversity within the community. So, the term “social–emotional learning” alone is enough to cause confusion.

There are over 136 frameworks that are thought to encompass over 700 SEL-related competencies [14]. As it stands, many of these frameworks focus on education and school-based implementations. There are a variety of often-cited programs that focus on both social–emotional skills and implementation, but there is a lack of studies that systematically define what constitutes social–emotional skills—something that is needed to establish meaningful developmental baselines. As a result, if one approach or program does not agree with the implementation recommended by a framework, then they may seek to create new boundaries of what social–emotional skills are as well as their own implementation recommendations. One of the largest SEL organizations, the Collaborative for Academic, Social, and Emotional Learning (CASEL), has a comprehensive framework that they suggest for SEL curricula. CASEL offers credentials through their organization based on a framework developed by CASEL called Transformative Social–Emotional Learning (T-SEL) [15]. They recommend a particular implementation for core competencies [16] then accredit programs in tiers, based on how close a program is to their framework.

Some studies speak of “social–emotional learning” as the field as a whole, while only citing CASEL’s framework [17,18,19,20]. This may possibly be due to CASEL’s name, “The Collaborative for Academic, Social, and Emotional Learning” [16]. Those who are not well acquainted with the field could assume CASEL’s approach of meeting all 5 competencies is required for social–emotional learning to happen developmentally. Their framework is a comprehensive approach, or a general set of methods and beliefs that many programs can fit into, rather than the exhaustive set of the diverse meanings of SEL from the literature within developmental psychology, cognitive neuroscience and social psychology, among others. CASEL’s framework is supported by extensive research, when it comes to efficacy. However, it is important that parents and policymakers realize that if the CASEL approach, or any SEL approach, does not work for their needs, there are many other options for approaches and programs to choose from. As discussed in more detail below, SEL does not have to be denied as a whole if one approach or program does not fit the contextual needs of a community. Since CASEL and other organizations like it have implementation requirements for their learning programs, which cater to a child or adolescent classroom setting, their framework does not extend outside the educational setting to the environmental demands of parent programs, therapeutic courses, and other places where social–emotional learning could benefit adults who are currently struggling with their social–emotional skills. An inclusive framework that finds overarching, simplified social–emotional themes and categories, which has no implementation barriers or formal mandates, could aid not only in making comparisons across program options, but it could also provide language for practitioners and educators to explain to clients and patients how basic skills develop.

As stated initially, the goal of this paper is to further the effort to create a taxonomy of SEL skills and the critical features of physical, social, and cognitive functions that support them. Once a unified framework for the development of social–emotional skills can be developed, then two important goals become easier. The first is the identification and evaluation of the natural strengths and weaknesses of individuals and the implications of those strengths and weaknesses on education and social–emotional needs. The second is that programs and approaches can be more clearly separated into modalities, with resulting improvements in the validity of outcomes assessments and modality comparisons.

## 2. Methods

A narrative literature review of scholarly, peer-reviewed articles was conducted using EBSCO Host, searching databases that included Academic Search Complete, Education Full Text, ERIC, Humanities International Index, MEDLINE, APA PsychArticles, APA Psychinfo, and Teacher Reference Center. It is narrative in nature because articles were collected with the focus of creating a psychological and developmental framework that addresses overarching themes. Due to the breadth of social–emotional skills and the research addressing them (thousands of published articles), research publications had to be sampled from many different fields including development, education, psychology, and sociology to generate a representative sample. The terms searched in all fields included “SEL”, “Social–Emotional Learning” and “Social Emotional Skills”. Our inclusion criteria were peer-reviewed empirical articles and published literature reviews, including systematic and meta-analytic papers. When literature surveys are used to create or modify a conceptual framework, there is the danger of inclusion/exclusion criteria inadvertently generating a confirmation bias for the model. To avoid this possibility, two approaches were used: 1. Regarding other reviews, as long as articles were published in recognized, peer-reviewed journals, and at minimum used terminology consistent with the literature, they were included. 2. Regarding empirical articles, since our goal was to provide conceptual bridges between areas of theoretical work, we generated a representative sample of the hundreds of articles that purport to empirically document facets of SEL. 

In addition to empirical papers, the aforementioned peer-reviewed literature reviews included meta-analyses, systematic reviews, and narrative reviews. References to commonly cited (i.e., popular) works were also included, but only when they reflected widespread views or goals regarding SEL programming. Our goal was to construct an interdisciplinary social–emotional skill framework that would be distinct from the vast literature on SEL implementation recommendations and requirements. This is for the primary reason that mapping out conceptual underpinnings of different terms and constructs from implementation literature can result in tautological (i.e., circular) conceptual justifications. However, the largest body of SEL research originates within the education field and often uses frameworks with an implementation focus. Therefore, this review does include some publications created with implementation-inclusive frameworks, when they incorporated detailed discussion or analysis of published empirical data.

## 3. Results

### 3.1. The Developmentally Integrative Hierarchy of Social–Emotional Skills

It is imperative to understand the basic development of social–emotional skills before attempting to compare and contrast different SEL programming. It is not within the scope of this paper to attempt a comprehensive overview of infant and childhood neurocognitive development that underlies social–emotional growth; but we address key areas that are critical to evaluating how SEL skills are defined and how programs attempt to support them. With a basic understanding of the development of social–emotional skills, we can compare and contrast how programs affect social–emotional skill development. 

Humans are social creatures, and in order to be socially successful, we need some competence in social–emotional skills. In an educational context, the main objective of social–emotional learning is to become capable of integrating into a community and working well with others. Therefore, within our model we have called this domain at the top of the hierarchy “Collaboration”, used broadly to encompass a range of social contexts. This is evidenced by the fact that when social–emotional skills are lacking, it results in disruptive behavior that hinders collaboration and negatively impacts the community. As a result, assessments are recommended to check for possible disabilities or delays. It is for this reason that when we speak about proficiency in a social–emotional primary skill, or a social–emotional competency, we are speaking about proficiency in a skill that will eventually lead to improvement in a person’s ability to collaborate.

**Figure 1:**
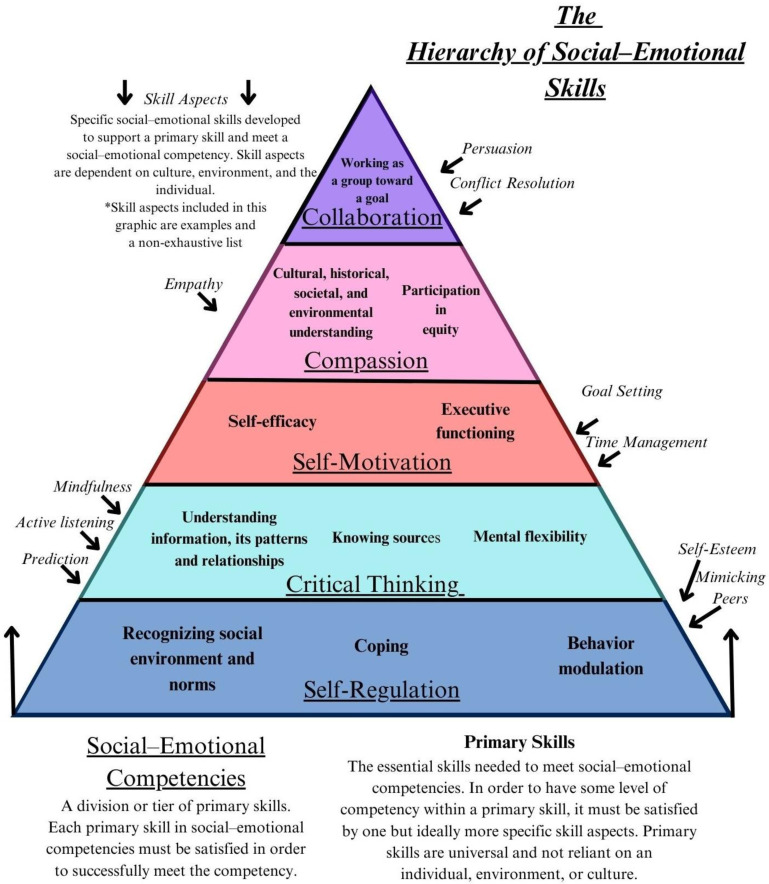
The hierarchy of social–emotional skills. Social–emotional competencies are underlined, primary skills are **bolded**, and skill aspects are *italicized* for clarity.

For the purpose of this framework, primary skills are grouped into tiers called social–emotional competencies, shown in Figure 1. The social–emotional competencies are as follows: Self-Regulation, Critical Thinking, Self-Motivation, Compassion, and Collaboration. These terms are capitalized to distinguish the competency as a part of our proposed framework from the generalized or popular use of these terms, which will be represented in lowercase. Due to polysemy, it is necessary to discern between the two. Primary skills within this framework are necessary for social–emotional development and the fulfillment of a social–emotional competency. Primary skills define a competency and are universal across cultures, environments, and individuals. All primary skills within a social–emotional competency must be met with at least one (but ideally more) of a number of skill aspects (i.e., facets) which make up the primary skills. While at least one skill aspect is necessary to each primary skill, there is no specific skill aspect or combination of specific skill aspects that are required to be competent in a primary skill. This is because skill aspects fluctuate based on environment, culture, or an individual’s needs. For instance, the two primary skills, *executive functioning* and *self-efficacy*, must both be met in order to have fulfillment in the social–emotional competency of Self-Motivation. The primary skill of executive functioning can include specific skill aspects such as delayed gratification or prioritizing, which both support it. Someone who can delay gratification but cannot prioritize is still showing some level of competency in the primary skill of executive functioning. They may live in an environment where prioritizing is done for them, so they do not have to learn this skill, and instead focus on other executive functioning skill aspects.

**Figure 2:**
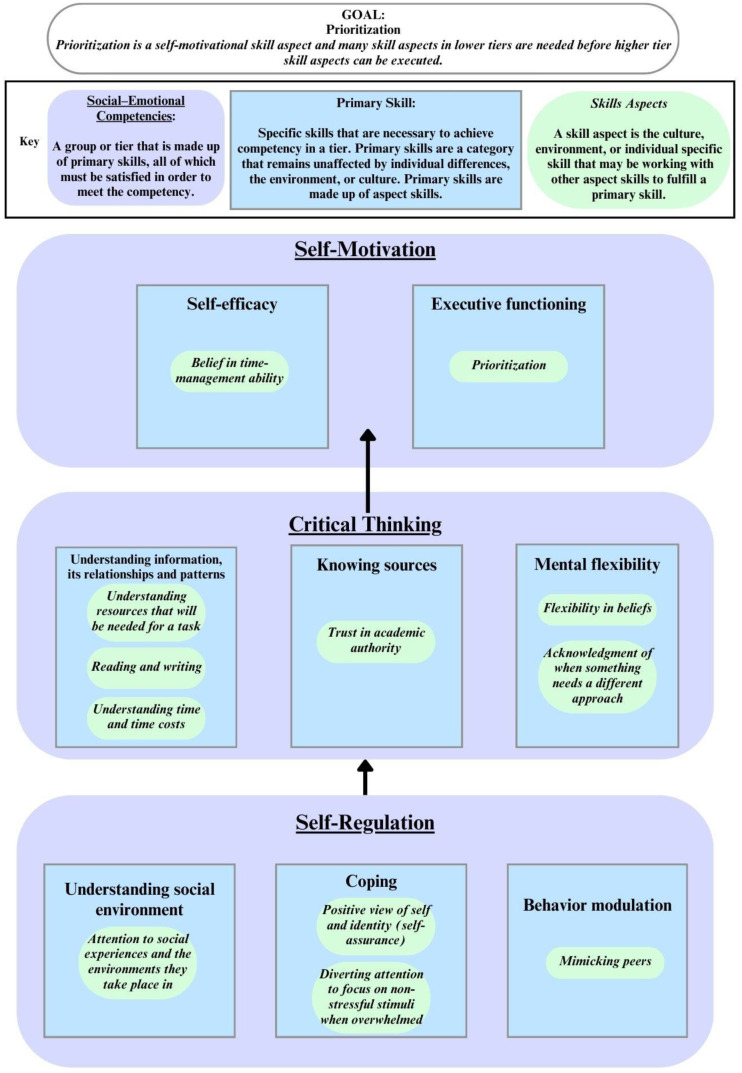
Example of how skill aspects build primary skills which build social–emotional competencies.

This specific skill aspect, prioritization, is a form of high-level executive functioning. Prioritization means understanding how to weigh the importance of tasks and perform them in the most efficient order. To prioritize well, one needs Critical Thinking aspect skills like recognizing time requirements, available resources, and the need for partners. These three Critical Thinking skill aspects support the primary skill of *understanding information* within the competency of Critical Thinking. When one combines Critical Thinking skills with the ability to follow through on tasks, which is an executive functioning skill, a person develops the higher-level skill aspect of prioritization within the social–emotional competency of Self-Motivation. Figure 2 shows a visual of this, but it is important to note that in real-life situations, an individual may utilize more skill aspects than what is shown in the figure. Each tier of social–emotional competencies in the hierarchy has its own set of skill aspects that also develop. In simpler terms, as one climbs the hierarchy, skill aspects become more complex.

However, a principle that geometrically complicates the picture of SEL is that most skill aspects and competencies emerge developmentally across levels within the nervous system and within higher-order cognition in the brain. As will be discussed in more detail below, there is a bidirectionality of influence among SEL abilities that only reveals itself ontogenetically. We can refer to a young child’s ability to emotionally self-regulate as being a prerequisite fundamental to high-level forms of executive function [21], such as time management within Critical Thinking. However, at later ages, within adolescent formal operational reasoning, critical thinking ability can be the moderating, rational voice that helps a teenager manage fear or anxiety (aspects of self-regulation). This bidirectionality of influence does not invalidate or contradict the taxonomies but simply requires one’s model of SEL to accommodate feedback loops among tiers of function. 

In our framework, there are five social–emotional competencies that derive from a systematic review of extant published research (see Section 2): Self-Regulation, Critical Thinking, Self-Motivation, Compassion, and Collaboration. It is critical from the outset to interpret these competencies as both hierarchical (i.e., lower areas form prerequisites for higher-order functions) and also internally multi-tiered (i.e., each area can be viewed as having high and low levels). Each following section will explain the competency in its most basic form, what it looks like when that specific skill is successfully used in the environment, and what it looks like when the skill is not successfully developed for the environment. 

### 3.2. Self-Regulation

In our proposed framework, the social–emotional competency of *Self-Regulation* focuses on the ability to have control over one’s own behavior and regulate it in a contextually acceptable manner. The meaning and scope of self-regulation has been debated for decades. Kaplan [22] mentions multiple difficulties in defining self-regulation, including implications of conceptual boundaries. For example, what may be potential sub-types of self-regulation are often argued to be the primary categories of self-regulation. He also mentions the importance of environmental context, as self-regulation is not a unitary, absolute construct (p. 483).

Furthermore, across different literatures (e.g., education vs. psychology) “self-regulation” is often used synonymously with “executive function”. In the developmental psychology literature, executive function is a construct that is immensely broad and multi-tiered, ranging from neonatal ability to visually and auditorily track targets [23,24] to the emergent ability in preschoolers to succeed at delayed gratification tasks, such as the famous Mischel [25] marshmallow test. In later childhood, adolescence, and early adulthood, the principle of executive function can expand to include self-monitoring, critical thinking, and eventually higher-order metacognitive reasoning. When using this framework as a lens to analyze previous studies, we can re-evaluate them within the context of distinct social–emotional competencies from our proposed framework only if we go beyond their conventional meanings. 

The concept of self-regulation, for the purpose of evaluating children in support of their social–emotional skills, needs to be clearly defined in order to implement coherent educational policy. For this reason, the base of our proposed framework (the Self-Regulation competency) is made of three measurable primary skills: the ability to recognize the environment and norms, coping, and behavioral modulation. Recognizing the features of the environment is a critically important primary skill of Self-Regulation. A common frustration among parents and educators is the apparent paradox: how can the recognition of norms be developmentally fundamental when it is such a complex ability? For infants, it is less complex. Using their senses and memory, they experience environments. The skill aspects infants are using to support *recognizing the environment* are things like touch, taste, and sensory information which are stored in memory. Recognition does not mean understanding, it only means to be aware of both the environment and the social actions taking place within it, such as found in the neonatal imitation of facial expressions [26,27,28]. Later, infants and young toddlers are able to notice more complex patterns of social behavior (e.g., peekaboo games) and develop expectations for social norms [29]. These are the low-level developmental beginnings of self-regulation. 

A second primary skill of Self-Regulation is emotional coping. While self-regulation is often considered an internal process, it is the external environment that an individual must navigate and interact with, which can be measured or socially observed [29]. While recognizing the environment is external, coping is primarily internal and individualistic. It is a skill of internally managing negative stress and finding the best way to meet needs while being appropriate in an environment. Sometimes a need cannot be met right away, such as in waiting for the bathroom or staying awake while driving. The longer the need goes unmet, the harder it is to ignore, and eventually, anyone would no longer be able to hold out and resort to immediately meeting the need. Coping can be an internal motivation to withstanding negative stress and mitigating negative behavior outcomes.

The third primary skill in the Self-Regulation competency is behavior modulation. Each environment will have a spectrum of acceptable behaviors and allowed forms of coping. Behavior modulation includes modifying all types of behavior, not just mitigating stress. Things such as curiosity, excitement, or a habit from a different environment, may result in behavior unacceptable to the new environment (e.g., “curious” biting behavior in toddlers or a preschooler tasting others’ food). The ability to mimic peers is an example of modifying one’s own behavior to match the social environment. This last primary skill, together with coping and recognizing the environment and its norms, creates the competency of low-level Self-Regulation within the base of the hierarchy. Once this competency is met, even on a very basic level, one can move to the tier above it, which is Critical Thinking.

However, as discussed earlier, there are often bidirectional feedback systems that emerge developmentally, where “high-level” functions like Critical Thinking can later moderate “low-level” functions such as emotional coping (within the Self-Regulation competency). The neurocognitive mechanisms for this kind of developmental feedback loop can be described as follows: well prior to adolescence, the development of the limbic system, particularly the function of the amygdala, is functionally mature, working to process and mediate environmental stimuli (e.g., potential threat, or sources of fear) via the hypothalamus–pituitary axis, which regulates the sympathetic nervous system. Because the amygdala is a structure that both processes threat/fear stimuli and encodes memories as part of a system of environmental adaptation [30,31], a child’s history of trauma would generate cognitive schemes for adapting to future threat and associated response sets (e.g., inhibition, defensive or aggressive responses to socially stressful encounters, etc.) [32]. The amygdala and associated areas of the limbic system are mature much earlier than areas of the ventromedial prefrontal cortex, which provide critical mediation of emotions, through higher-order (i.e., cortical) information processing directly related to critical thinking (objective risk assessment, self-monitoring, etc.) During typical development in adolescence, prefrontal cortical function begins to “catch up” with evolved systems for threat detection and response, bringing into balance a person’s ability to use objective evaluation with natural threat response ability [33]. Normal (i.e., non-trauma history) childhood experiences that support healthy responses to anxiety set the path for later healthy development of critical thinking, which in turn feeds back to lower-level emotional self-regulation when occasional periods of stress or threat emerge. As a result of this temporal relationship between the limbic structures and key areas of the prefrontal cortex, traumatic childhood experiences that are chronic and/or severe (which in themselves have unique and often unpredictable sequelae) can generate patterns of later dysregulation that inherently make systemic corrections (i.e., behavioral adaptations) difficult. 

The definition of Self-Regulation within this framework validates how emotional dysregulation is connected to the cyclical nature of trauma [34]. An example of the cyclical nature of trauma is that a parent who has experienced partner violence is more likely to have a child that also experiences partner violence. One who is used to a particular environment may have more coping skills and more experience with modifying behavior relevant to that specific environment. A new environment, even if it is safer, will have different stressors and different socially acceptable coping skills or norms of behavior, making self-regulation more difficult for that individual despite it being a “safer” environment. Children accustomed to living in a household where partner violence exists may feel less stress in a future similar partnership because they know what to expect and what coping skills to enact. They may feel more “goodness of fit” in an abusive relationship where they know how to avoid abuse through apologies and meek behavior instead of having to learn a new environment, coping, and behavior modification techniques altogether.

With the emergence of brain imaging technology applied to social–emotional development, we have come to appreciate the organic nature of SEL and the areas of great vulnerability. For example, recent natural experimental studies [35] of children who have endured extended, neglectful institutional environments (i.e., orphanages and unstable foster care) have found a linear relationship between amygdala size and activation (our brain’s organ for evaluating and categorizing threat) and actual performance on tests that evaluate speed and accuracy when responding to neutral, negative, or friendly faces. Children with abuse history and/or who have endured long periods in institutional care are far faster and more accurate in labeling negative affect faces compared to control children. In other words, in the context of our argument for contextual sensitivity, these children have developed neurocognitive SEL adaptations that are highly advantageous in an insecure, even dangerous, environment but are disastrous in a “normal”, safe educational or residential environment. Sadly, they tend to be slower than study control groups to label faces as friendly, and they are inaccurate when labeling neutral faces, tending to default to “negative” [36].

Similarly, according to Thomaes [37], abuse victims have significantly less gray matter in the orbitofrontal cortex compared to controls. The orbitofrontal cortex is linked to cognitive processes such as decision-making, which is integral to critical thinking. Interestingly enough, this same study reported reduced gray matter in the anterior cingulate cortex which aids in executive functioning and empathy [38]. Abuse victims have to be focused on perceiving their environment in order to meet the basic need for safety, and they may struggle to move up the hierarchy to Critical Thinking, Self-Motivation, Compassion, and Collaboration in a proficient manner in a safe environment because of it.

### 3.3. Critical Thinking

Critical Thinking (also known as higher-order reasoning or complex cognition) in our proposed framework focuses on the ability to objectively analyze and evaluate information in order to form a judgment or make a decision. The definition of critical thinking has less controversy surrounding it, and the term is used less flexibly in the literature. The epistemological goal of critical thinking is to assess the quality and validity of information and its relevance to making real-world judgments [38]. It is often associated with the academic portion of schooling children. The first primary skill that makes up the social–emotional competency of Critical Thinking in our proposed framework is understanding information, its patterns, and relationships. The second primary skill of Critical Thinking is recognizing and evaluating source validity. The third primary skill of Critical Thinking is mental flexibility.

The first primary skill of Critical Thinking is understanding information, its patterns, and relationships. As discussed earlier regarding Self-Regulation, it was mentioned that recognizing information and understanding information were two different things. Recognizing contextual information is when someone encounters information multiple times and acknowledges the connection of place, time, or existence on a surface level. In infant development research, this is referred to as assimilation. For example, in early infancy, an exogenous (i.e., “social”) smile may occur in response to the sound of mother’s voice, which is a form of neurocognitive assimilation [39]; a later example would be when a toddler notices that the family is speaking louder when visiting Grandma’s house and complying with the norm. In Self-Regulation, one recognizes the norm to speak loudly at Grandma’s house. Understanding information requires having insight that Grandma cannot hear well, and it is specifically Grandma that people are speaking to more loudly. For the toddler, it has become a specific accommodation rather than a basic social or behavioral norm. This takes time to develop in children. Understanding information, its patterns, and relationships is a complex undertaking that a child’s academic schooling will typically focus on developing and improving.

The second primary skill of Critical Thinking is the recognition and evaluation of sources, to be able to evaluate the accuracy of the information they receive and to determine their confidence level in their judgments. A low-level developmental example is a child trusting their parents because they are the source of comfort and care. In schooling, they may trust their teacher because their parents trust their teacher; or they may trust a teacher because the majority of their peers trust their teacher; or they may not trust the teacher until their own relationship is forged with them. A higher-level example of critically evaluating sources would be the credentials of a person, citations in research, and the acknowledgment of earned prestige.

The third primary skill of Critical Thinking is mental flexibility. An individual must remain receptive to new ideas, perspectives, and insights while striving to understand the nature and relationships between different pieces of information. This may mean flexibility in the sense of replacing outdated ideas or gaining new insight about sources that may be no longer credible. It also means mental flexibility in the way of understanding information in a less concrete way, recognizing information in theory vs. practice, symbolism, and hypotheticals.

Although complex, one does not have to have mastery of all the primary skills in order to move up the hierarchy. Only basic competency is necessary for each primary skill of the Critical Thinking competency to move up the hierarchy to begin learning the primary skills within the Self-Motivation competency. Figure 2 shows an example of how skills are learned and built into executive functioning, a primary skill of Self-Motivation. Some researchers propose that critical thinking is a component of executive functioning. However, in our framework, Critical Thinking is treated as a distinct social–emotional competency due to its unique social objectives compared to executive functioning as defined in the section titled “Self-Motivation” in this paper. Executive functioning aims to control, coordinate, and integrate tasks for efficient performance, whereas critical thinking focuses on making judgments and solving complex issues [39]. Despite being considered a separate competency, Critical Thinking is essential for executive functioning as it operates hierarchically below it. In fact, executive functioning relies on the existence of Critical Thinking and Self-Regulation at a low level before it can fully develop.

However, returning the principle of bidirectionality, it is also argued that Critical Thinking can enhance Self-Regulation. On a fundamental level, some level of Self-Regulation is necessary to engage in Critical Thinking, as seen in the ability to suppress a natural fight-or-flight responses (e.g., suppressing the “prepotent response” of panic in order to safely perform a task requiring objective evaluation). However, the interplay between these different skills can be intricate and sometimes confusing. As social–emotional skills become more sophisticated, they still build upon the foundation of simpler skills learned hierarchically. Even complex skills follow this natural hierarchy. For instance, individuals can employ Critical Thinking skills (e.g., objective risk evaluation) to achieve more advanced Self-Regulation techniques (e.g., suppressing fear of strangers in situations requiring one to meet new people). In an academic context, mindfulness (a facet of metacognition) may involve effectively budgeting time and prioritizing subtasks—activities that require Critical Thinking. When mindfulness facilitates successful Self-Regulation in fulfilling tasks, especially multi-step ones, it becomes part of the Self-Motivation competency, discussed in the next section.

In this case, the goal of the Self-Motivation skill is to enhance Self-Regulation in a feedback loop. One might wonder why Self-Regulation is a goal when individuals already possess a foundation of it in order to be functioning at the level of Self-Motivation. The answer lies in the need to strengthen or diversify skills at lower competency levels before progressing to higher competencies, emphasizing the hierarchical structure. For individuals juggling numerous tasks, the ability to critically think about aspects like time and resources while completing these tasks and having high Self-Regulation becomes vital. Building higher skills necessitates reinforcing lower ones, establishing a solid and well-rounded social–emotional skill set.

When there are failures in Critical Thinking, which may originate with difficulties in low-level Self-Regulation, then key areas within education, such as task execution, connecting with others, or collaborative tasks, often lead to a state of emotional dysregulation (i.e., a critical thinking deficit can belie a state of fear or anxiety). In order to recover from this, one needs to ascend the hierarchy once again. However, a cruel Catch-22 is that this often requires skills that were compromised in the first place: regulating, engaging in Critical Thinking to devise a new plan, effectively executing tasks through Self-Motivation, and establishing sufficient connections with others to respond appropriately in Collaborative efforts.

### 3.4. Self-Motivation

Self-Motivation in our proposed framework focuses on the ability to drive oneself or take the initiative to pursue goals and complete tasks. As such, it requires emotional strengths and self-management, combined with facets of executive function. Whereas Self-Regulation has the function of making behavior blend in and meet social norms, Self-Motivation is focused on making behavior productive for the individual. Self-Motivation allows one to become self-sufficient and work independently in the individual’s best interest. The two primary skills in the Self-Motivation competency include self-efficacy and executive functioning. 

As discussed, the primary skill of executive functioning has had many different definitions from simply delaying gratification to understanding consequences and future goals [39]. High-level executive functioning in this framework is considered to be the ability to efficiently create reasonable goals, take steps to meet them, and reduce behaviors that distract from meeting them. Skills that would aid in this include inhibitory control, working memory, and mental flexibility. Inhibitory control can be things like controlling impulses, controlling conditioned responses, and delaying gratification [40]. Working memory (also known as short-term store) has a bidirectional relationship with attentional processes, defined as the ability to attend to information in one’s mind while working out a problem. It is essential for briefly holding units of information (e.g., allowable chess piece movements) steady in mind, while evaluating how best to create a sequence of steps to further a goal (e.g., counter a chess opponent’s move). Conversely, conscious, deliberate attention (e.g., attending to categories and rules), as a facet of executive function, is precisely what is required to maximize the retention of information in working memory. Things such as organizing, prioritizing, and multitasking depend on working memory; and working memory, in part, can depend on intact attentional processes.

Mental flexibility in executive functioning literature is often described as the ability to transition between tasks or modes of thought [41], whereas mental flexibility in the critical thinking literature is often the ability to evaluate information and change one’s mind when new information comes to light [38]. Mental flexibility as a primary skill of Critical Thinking is necessary in our framework, whereas mental flexibility within executive function is a skill aspect focused on transitioning tasks. Transitioning tasks is important, but someone who struggles with transitioning tasks can still display executive function in a low-level form of executing a single task. 

The second primary skill within Self-Motivation is self-efficacy, which is the belief in one’s own capacity to execute a task. It takes both actual ability and belief in ability in order to execute a task. Without some belief in ability, one would not attempt the challenge. Those who have had ample opportunities to master social–emotional skills will have belief in their skills going forward to collaborate and also may pick more difficult social challenges (e.g., initiating new friendships at a party), giving the individual a more complex history of practice [42]. Watching others succeed can form a vicarious experience and is one important reason behind making sure children have role models for social competence to project themselves onto, since seeing someone similar to you succeed can boost confidence in your own ability [43].

An interesting example of how Self-Motivation affects higher level competencies (Compassion and Collaboration) can be found in ADHD (attention deficit hyperactivity disorder). Children who have ADHD, a disorder defined by symptoms that disrupt executive functioning, have very well-documented social deficits in collaboration [43]. Although research shows children with ADHD display lower empathy (a skill aspect strongly linked to compassion), the emotional intensity and reactivity (aspects of self-regulation) in these children are similar to controls of children without ADHD [44]. This framework explains how children who struggle with Self-Motivation, whether with ADHD or not, struggle with higher SEL levels of Compassion and Collaboration. However, they do not necessarily struggle with emotional regulation or critical thinking. While there have been studies that find emotional dysregulation to covary with ADHD [45,46], it is important to point out that teachers and parents report higher levels of emotional dysregulation, whereas professional observation shows mild effects if any. This may indicate that the evaluated ADHD children are not given proper accommodation due to their caretakers’ lack of knowledge or lack of resources causing emotional dysregulation in the children. If teachers and parents are frustrated by their children’s lack of functioning, this could also affect the environment in which the children are being evaluated. Professionals who are knowledgeable about the disorder can give proper accommodation to children while evaluating them [37]. So, it could be likely that ADHD does not have a characteristic of emotional dysregulation in and of itself.

### 3.5. Compassion

In this SEL framework, Compassion at its most basic, fundamental level, is the ability to predict and recognize dysregulation in others and take steps to prevent or alleviate dysregulation. This can improve with guided, cognitively based interventions [47]. Like self-regulation, compassion has some controversy in regard to its definition [48], often centering around its dual manifestation as an internal state of mind vs. its behavioral and social appearance [49]. Generally speaking, though, most agree that compassion is to be emotionally moved by others’ suffering, to want to take action, to alleviate, or to prevent suffering. Our proposed framework is primarily focused on the measurable aspects of social–emotional skills, which are essential for research and ultimately so that implementation of educational policy and SEL programming can be evidence-based. The first primary skill of Compassion is understanding cultural, environmental, historical, and social structures. The second skill is participation in equity.

Compassion often depends on cultural, environmental, historical, and social structures; thus, on a fundamental level, compassion might be argued to be perceptual and cognitive in nature, rather than a purely emotion-driven sensitivity. In Compassion, an individual attempts to recognize a diversity of people’s lived experiences, histories, cultures, social structures and use that to predict or alleviate dysregulation among individuals. This is a mix between recognizing and understanding the environment, which exists in Self-Regulation, and Critical Thinking, again highlighting the structure of the hierarchy. Some basic level of meeting each of the primary skills below Compassion is required to practice Compassionate skills. 

The second primary skill within Compassion is participation in equity. We have coined this expression to capture the concept of collective motivation to share equally in an experience—both at the contribution level (e.g., working together) and the outcomes level (sense of collective achievement). Participation in equity is any behavior or communication choice that serves to make the social or physical environment safe, accessible, and welcoming to others. It is a choice one makes specifically to share regulation with others, so one can then move to Collaboration. Unlike the more common connotation of equity as relating to fairness, equity is not merely a construct contrived to accommodate needs of a minority group. We are using the term to capture social outcomes that occur collectively, when individuals show the capacity to accommodate as a general frame of mind, or a disposition. It happens when you bring your grandmother a plate of food because she cannot walk or remove a rock from a two-year-old’s hand because he does not know that it is a choking hazard.

Participation in equity can also be engaging in group equity. One may speak louder to a cousin who lost his hearing, and the cousin may lean forward cupping his ear to show that he is doing his part to hear the speaker and collaborate in communication. Group equity is two or more people engaging in finding a middle ground to collaborate. This is performed with the knowledge or estimations they have about their group members and their experiences. If an environment exists that is not accommodating and an individual cannot regulate and adapt to it, then setting boundaries and removing themselves is the Compassionate act. However, the greater the proficiency in Compassion, the less those with higher needs have to remove themselves or not participate, which leads to all of the creativity and success that comes from diversity [50,51]. Participation in equity, in its ideal form, can be a collective state of SEL functionality that can distribute accommodative burden across all its members.

For some, equitable behavior and collaboration are second nature, or highly socialized; but there are those who struggle with any number of the many prerequisite competencies and find it difficult. Low-level compassion can be almost effortlessly accommodating someone only slightly different than yourself. In rural areas with minimal cultural diversity, that may be all that is necessary. In areas with low diversity, children may not encounter opportunities to practice the skills of interacting with diverse needs and/or may see very little role modeling of these skills. 

### 3.6. Collaboration

Collaboration in our model is positioned at the top of the hierarchy because in social, educational, and work environments, it represents a fundamental level of organizational functionality. The definition of Collaboration is working with two or more people toward a goal. A low level of proficiency in the four former competencies is required to Collaborate. The competency of Self-Regulation focuses on controlling and regulating one’s own behavior in an environmentally acceptable manner. Critical Thinking is required to objectively analyze and evaluate information to form a judgment. Self-Motivation entails the ability to direct oneself or take initiative to pursue goals and complete tasks. Compassion enables one to recognize and predict dysregulation in others and take steps to prevent or alleviate dysregulation in the group so they will be willing to work together. The only primary skill of Collaboration is efficiently working as a group towards any goal. Self-Regulation, Critical Thinking, and Self-Motivation are intrapersonal skills, whereas Compassion and Collaboration are social skills.

Collaboration, as a state of individuals participating in equity, can manifest in many ways. A low-level example would be strangers taking turns to walk around a barrier on a narrow sidewalk, rather than both continuing and crashing together, or putting either at risk of walking on a street where a car might hit them. A high-level example of Collaboration in language can be seen in poor rural areas where a particular slang vernacular exists. In TV and films, rural people are often portrayed as ignorant or uneducated because of their speech. In reality, shortening and creating more emotive and direct language gets communication across in less time, with more power. The use of swearing to succinctly convey passion versus indifference in a sentence can be a sophisticated form of brevity. A single mother of four, a college student who also works, or a man with three jobs still has social needs but does not have adequate time to learn magniloquent vocabulary to express oneself or have much time to meet their social needs. Instead, these areas have adapted to be able to have quick and passionate conversations which are easily understood to convey information. Shorter, more intense language being agreed upon as acceptable in the community is a complex form of Collaboration, as we have defined it here.

## 4. Discussion

### 4.1. Using the Developmentally Integrative Framework for Comparisons

The hierarchical framework we proposed provides a conceptual taxonomy on overarching themes that exist in the diverse scholarly social–emotional skill literature. By integrating neurocognitive, developmental, and educational perspectives that relate to SEL, we created a framework that can be used in a variety of different ways. For example, this framework explains social–emotional behavior in a de-pathologizing way, and it does so without recommending implementations or ways to change behavior. The primary skill of recognizing the environment within the Self-Regulation competency is at the base of the hierarchy because it is a fundamental social skill, emerging in early infancy that must be developed for the environment one is in. There are times where social skills do not match well to the environment which is not only seen in children but also adults. For instance, this mismatch can be seen in adults growing up in poverty transitioning to the middle class, combat veterans coming back to civilian life, or immigrants with a vastly different culture coming to a new country. Having an integrative explanatory framework allows clinical practitioners and educators to look at what domains they perceive individuals to be struggling in and, importantly, draw on an integrative model that may alert them to lower-level antecedent difficulties. This more integrative perspective can enable them to make the decision as to whether they should continue to adhere to their culturally derived models of care or support their clients or students to learn new skills to see if it helps them adapt. While this model cannot recommend how those new skills are learned, it gives practitioners an idea about the domain and types of skills they should focus on. The other way this framework can be used is to compare existing SEL programming that has implementation, skill diversification, and behavior modification recommendations. Currently there is confusion from educators and parents as to what SEL is and how programs differ; this framework allows for comparison and clarity. We have given an example of this below.

#### 4.1.1. CASEL T-SEL

The CASEL organization has created the most extensive collection of SEL literature and are well cited and esteemed in the field as a whole. The CASEL framework outlines recommended implementation and best practices for the most comprehensive SEL approach in this list. CASEL’s approach is inclusive of all five tiers of social–emotional development in our framework, which aligns with their whole child, whole school, and whole district beliefs. 

#### 4.1.2. Collaborative/Cooperative Learning

Cooperative learning is a skill-specific approach that focuses on collaborative skills that are taught through the repeated practice of group academic sessions and activities [52]. While both collaborative learning and cooperative learning are used interchangeably, collaborative learning is sometimes referred to as when a group works together for an entire project, while cooperative learning is when each person does their part of a project and comes together at the end. Both approaches’ main focus is Collaboration or having a goal that individuals reach together.

#### 4.1.3. Trauma Informative Teaching

Trauma Informative teaching theory focuses on creating a welcoming environment through Compassion, Collaboration, and Self-Regulation. The focus on equity and community is typically bolstered by ways of regulating difficult emotions to improve community conversations [53,54].

#### 4.1.4. Mindfulness-Based Practices (MBP)

Mindfulness-based practices may appear to be Self-Regulation focused but involve Critical Thinking as well. Taking in information about how time works, the present, past, and future, and recognizing what is not relevant to the present moment is a form of Critical Thinking. Some aspect skills of Critical Thinking include re-framing and perspective-taking which are often used in mindfulness-based teaching practices [55]. For this reason, MBP focuses on skill sets of Self-Regulation and Critical Thinking.

#### 4.1.5. Flipped Classroom

A flipped classroom creates a focus on applying knowledge in order to deeply understand it rather than having a professor lecture the information. Students learn the initial work before class and then apply it through activities or practice during class time, typically in groups. The professor is available to work out issues only if it is needed. For this reason, flipped classrooms focus on skills of Critical Thinking to understand information, Self-Motivation to do the work before the class, and Collaboration to work with others toward the goal of learning [56].

#### 4.1.6. Montessori

The Montessori approach focuses on the skill of Self-Motivation by allowing children to guide themselves at a young age in the classroom with minimal adult guidance or lectures. It also focuses on giving resources that are sized properly for children to critically think and easily practice motions at their own pace. While this typically leads to Compassion and Collaboration, the focus of the Montessori approach is on supporting the student by letting them self-motivate and critically think about what they would like to do and why [12,57].

#### 4.1.7. Self-Directed Learning (SDL)/Self-Regulated Learning (SRL)

SRL pushes students to work with their own strengths and weaknesses to become driven and successful learners with the ability to adapt to their environment [58]. SDL is used interchangeably with SRL; however, some consider Self-Directed Learning to be a student coming up with their own goals and motivations without needing outside guidance, while Self-Regulated Learning has outside guidance, but the student finds their own ways to meet roughly set standards and goals from the teacher or institution. This gives SDL a methodology or teaching style difference while still existing within the same core skill sets as SRL. These skill sets are Self-Regulation for adapting to the environment, Critical Thinking to understand information and how to work with oneself to set goals, and Self-Motivation to follow through on those goals [59].

### 4.2. Educational SEL Program Comparisons

Since our proposed framework has an explanatory focus rather than an implementation focus, it only categorizes programs based on the skill categories it fits into. An approach may have many different programs that claim to be a part of the approach. What changes program to program are the aspect skills that are being taught. Hypothetically, if two programs were teaching the same aspect skills but had different forms of implementation, it would be easier to see which implementation resulted in the most positive outcome. Our framework allows for a basic evaluation of which SEL programs are focused on particular areas and also allows one to break down programs by specific skills. This is important for cultural and environmental context, as parents can look at what primary skills their children seem to be struggling with and look for programs that teach aspect skills that are appropriate for their culture and environment. It also allows populations currently wary of SEL programs to pick very specific options to “test the waters”, so to speak, rather than having to reject all of SEL as a whole because they are concerned about large changes to the school system.

It is important to remember that approaches with suggested implementations in education may not work well in mental health, social work, and community-building fields, either due to developmentally important differences between populations, or because of culturally important needs. Our proposed framework would also be able to compare and contrast things such as CPS reformation parent programs, trauma and abuse recovery programs, or addiction recovery programs as well. Specifically, this model can allow patients to look for programs that focus on lower tiers (Self-Regulation, Critical Thinking, Self-Motivation) or higher tiers (Compassion and Collaboration) depending on where they are in their journey of healing. Programs can be categorized broadly into these five tiers or become more specific by focusing on aspect skills which change based on environment or culture.

There are also times where social–emotional skills are helpful for the individual but not what an institution that seeks to implement SEL programming desires. There has been some concern in the general population that social–emotional learning programs reduce “grit” in children or make them “softer”. This argument, that SEL support reduces resiliency, depends on what “resilience” means in the context of the argument. If the term resilience means to be tough in the face of environmental threats, then this returns us to a misuse of the Darwinian principle of being “fit” and tacitly accepts inequity and poverty among communities, because it is being used to suggest that individuals should learn to tolerate abuse or environmental deprivation. When children learn things such as boundaries and how to self-advocate, this may feel inconvenient to caretakers and educators, and it may be tempting to blame the child for being ‘soft’ or ‘insubordinate’ for knowing their own limits. However, preventing abuse also prevents future mental health issues as abuse is linked to a wide variety of physical and mental health problems [11], so children who refuse to tolerate abusive behavior may be able to self-regulate better than the peers who withstand abuse. Along those same lines, while a school may benefit from children who collaborate well, if the district is in a very low-income area, they may need to focus on basic needs and resource seeking and not on higher tiers of Compassion and Collaboration. A pervasive pattern is that schools focus on forms of Critical Thinking, because, depending on the age level, critical thinking deficits have the most direct, visible link to academic difficulties but may hide more profound struggles with Self-Regulation, which is at the base of the hierarchy.

How could one evaluate how students are doing in Self-Regulation, Critical Thinking, Self-Motivation, Compassion, or Collaboration? As this is a theoretical framework, a survey does not yet exist and would need to be tested empirically. However, the breadth of definitions for each of these tiers allow for teachers to make theoretically inclusive evaluations of need. If a student is screaming, throwing, fighting, or crying, this would likely be Self-Regulation. If a student needs constant instruction, thinks in an overly concrete manner, or naively believes every source they get information from, this would most likely be Critical Thinking. If a student struggles with feeling as if they are so incapable that they refuse to try, or with starting, maintaining, or completing a task, this is most likely Self-Motivation. If a student is unable to change their behavior based on different social situations or specifically avoids students who are different from them, this is most likely Compassion. If a student will only work with others who do what they demand or if a student refuses to participate with other students involved altogether, this is most likely Collaboration. If an educator notices a particular area which children seem to struggle with, say Compassion and Collaboration, they can use our proposed framework to look at SEL approaches which typically focus themselves in these areas; in this case it would be the Trauma Informative approach.

**Figure 3:**
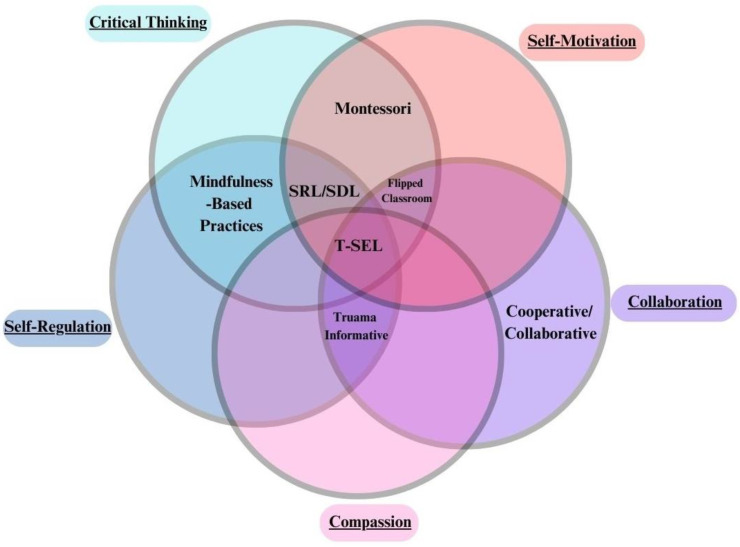
Educational SEL program comparisons. Social–emotional competencies are underlined  for clarity.

Skill aspects are specific skills that change depending on the environment or culture. There are a great many different skill aspects which exist. Some examples are the ability to name emotions your body is feeling (Critical Thinking), the ability to associate facial expressions to emotions someone else might feel (Compassion), listening skills (Critical Thinking), breathing techniques when getting overwhelmed (Self-Regulation), distraction when getting overwhelmed (Self-Regulation), and changing methods when a task has failed (Self-Motivation). While each skill aspect is connected to a distinct tier, the hierarchy means that in order to have reached that tier, the base skills below it helped in its creation. Changing methods when a task has failed includes regulating through the failure (Self-Regulation) and Critical Thinking to create a plan that is more effective before the final act of actually changing the methods and carrying out the new task after the old method has failed (Self-Motivation). The reason why this skill is considered specifically Self-Motivation is because of the hierarchical structure of social skills. One needs skills of Self-Regulation and Critical Thinking before a Self-Motivation skill can be developed. This is why in Figure 3, illustrating comparisons of SEL approaches, the program is sorted based on the skills that the SEL approach focuses on. For example, Montessori focuses on Critical Thinking and Self-Motivation in its recommendations. Self-Regulation is not being focused on, although one does have to have some skills in that area in order to move up to the higher hierarchies.

## 5. Conclusions

This framework serves as a tool to improve the understanding of social–emotional learning (SEL) for educators, policy makers, and parents. It provides structure to the varied social skills and social–emotional concepts across varying fields, and the hierarchical connection explains why researchers in these fields struggle with ambiguity in both definitions and terminology. While this framework only seeks to synthesize the SEL literature in many fields and structure social skills based on the overarching concepts and themes within that research, it can also be used for other important purposes. It implies that clinicians and educators should first identify perceived issues and then trace them to fundamental or latent areas of difficulty that may be very low level, rather than try to correct deficiencies at too high a tier by failing to see prerequisite needs. It also suggests to parents in particular that strengthening primary skills through the acquisition of specific skill aspects can enhance their child’s social–emotional development. Furthermore, it allows adults adapting to new environments and cultures to evaluate their own skills and see where they can make changes and diversify skills.

The most pressing future direction would be testing the hierarchy for usefulness in an education setting, CPS or parent reformation program, or therapeutic community settings, as a tool. A limitation of this paper is analyzing the 700 SEL skills which, in this framework, mostly exist within aspect skills [14]. This is both due to the limited size of the study and also because skills may be used differently based on culture and environment. So. it would be valuable to explore these issues in future research. Another limitation is looking specifically at programming skill aspects as most programs do not share their SEL curriculum without either payment or long teaching sessions. Considering SEL programs are often marketed toward schools and organizations, in the future, a collection and comparison of these programs would be beneficial. As more SEL research is inclusive of adults, community/cultural issues, and not solely educational contexts, this framework can aid in evaluating, comparing, and contrasting programming without implementation barriers. With consistent definitions and a fundamental understanding of the natural progression of skill-building in social–emotional learning, this framework lays a solid foundation for future research, education, and policy development.

## Data Availability

No new data were created or analyzed in this study. Data sharing is not applicable to this article.
